# A novel nonsense *PTH1R* variant shows incomplete penetrance of primary failure of eruption: a case report

**DOI:** 10.1186/s12903-019-0944-9

**Published:** 2019-11-15

**Authors:** Cristina Grippaudo, Concetta Cafiero, Isabella D’Apolito, Agnese Re, Maurizio Genuardi, Pietro Chiurazzi, Sylvia A. Frazier-Bowers

**Affiliations:** 1 Istituto di Clinica Odontoiatrica, Università Cattolica del Sacro Cuore, Fondazione Policlinico Universitario “A.Gemelli” IRCCS, Roma, Italy; 2 Istituto di Fisiologia Umana, Università Cattolica del Sacro Cuore, Roma, Italy; 3 Istituto di Biologia Cellulare e Neurobiologia, Consiglio Nazionale delle Ricerche (CNR), Roma, Italy; 4 Istituto di Medicina Genomica, Università Cattolica del Sacro Cuore, Fondazione Policlinico Universitario “A.Gemelli” IRCCS, Roma, Italy; 5 Department of Orthodontics, School of Dentistry, University of North Carolina, Chapel Hill, NC USA

**Keywords:** Primary failure of eruption, Orthodontics, *PTH1R* gene, Nonsense variant, Incomplete penetrance, Case report

## Abstract

**Background:**

Aim of this work was to describe a rare inheritance pattern of Primary Failure of Eruption (PFE) in a small family with incomplete penetrance of PFE and a novel nonsense *PTH1R* variant.

**Case presentation:**

The proband, a 26 year-old man with a significant bilateral open-bite, was diagnosed with PFE using clinical and radiographic characteristics. DNA was extracted from the proband and his immediate family using buccal swabs and the entire *PTH1R* coding sequence was analyzed, revealing a novel heterozygous nonsense variant in exon 7 of *PTH1R* (c.505G > T). This variant introduces a premature stop codon in position 169, predicted to result in the production of a truncated and non-functional protein. This variant has never been reported in association with PFE and is not present in the Genome Aggregation Database (gnomAD). Interestingly, the c.505G > T variant has also been identified in the unaffected mother of our proband, suggesting incomplete penetrance of PFE.

**Conclusions:**

In this study, we report a new *PTH1R* variant that segregates in an autosomal dominant pattern and causes PFE with incomplete penetrance. This underlines the diagnostic value of a thorough clinical and genetic analysis of all family members in order to estimate accurate recurrence risks, identify subtle clinical manifestations and provide proper management of PFE patients.

## Background

Primary Failure of tooth Eruption (PFE - OMIM #125350) is a nonsyndromic condition that is likely the result of multiple genetic and environmental factors that control tooth eruption [1]. PFE is characterized by incomplete eruption of teeth despite the absence of mechanical obstruction and the appearance of a normal physiological bone resorption along the eruption path [2–4]. It is variable in terms of the number and type of teeth involved (primary or permanent) and the degree of symmetry but only affects posterior teeth. PFE is defined by an apparent cessation of eruption after the tooth has emerged to a supracrestal position [5]. Typically, all teeth distal to the most mesial affected teeth are affected [6]. The most challenging aspect of eruption disorders is determining the specific cause and therefore applying the appropriate intervention. The similar clinical presentation of other eruption disorders such as those caused by a mechanical obstruction or mechanical failure (MFE), isolated ankylosis characterized by infraocclusion, immobility or severe delay of eruption, must be excluded [7, 8].

Several PFE cases have been associated with haploinsufficiency of the *PTH1R* gene (i.e. presence of only one functional copy of the gene), located on chromosome 3p21.31 (OMIM *168468). The presence of a loss-of-function variant in *PTH1R* confirms the clinical diagnosis and is associated with an autosomal dominant pattern of inheritance. Affected individuals are therefore heterozygous possessing one normal copy (allele) and one non-functional allele of *PTH1R* [9, 10].

More than 50 different *PTH1R* variants have been reported in PFE patients, distributed through its entire coding sequence and causing protein loss-of-function [8]. Affected individuals carry one normal copy (allele) of the *PTH1R* gene and a variant allele with a missense substitution, a nonsense (stop codon) change resulting in a truncated protein or a frameshift variant resulting in a protein with an abnormal C-terminal domain [9, 10]. Half of the receptor activity is apparently enough to compensate for PTH1R function in most tissues; however, haploinsufficiency may not be always enough to allow normal tooth eruption, determining PFE with incomplete penetrance i.e. PFE may not be present in individuals who carry a loss-of-function *PTH1R* variant [11]. In other cases a PFE patient has no family history since he/she inherited a de novo mutation from the germ cell of either parent. It is therefore important to determine if an unaffected parent of a PFE patient is a non-penetrant carrier of a *PTH1R* variant since he/she could have more than one affected child. In any case, establishing a genetic diagnosis is extremely important before planning any orthodontic treatment, considering the high failure rate of orthodontically assisted eruption in individuals with PFE [5, 12].

In this report we present a patient with severe PFE who was found to carry a novel *PTH1R* variant (c.505G > T; p.Glu169Ter) that has been transmitted by the unaffected mother, providing a typical example of incomplete penetrance. We therefore posit that even in isolated cases of PFE, the genetic analysis offers a valuable metric to predict the potential success of orthodontic intervention.

## Case presentation

### Clinical analysis

**Fig. 1:**
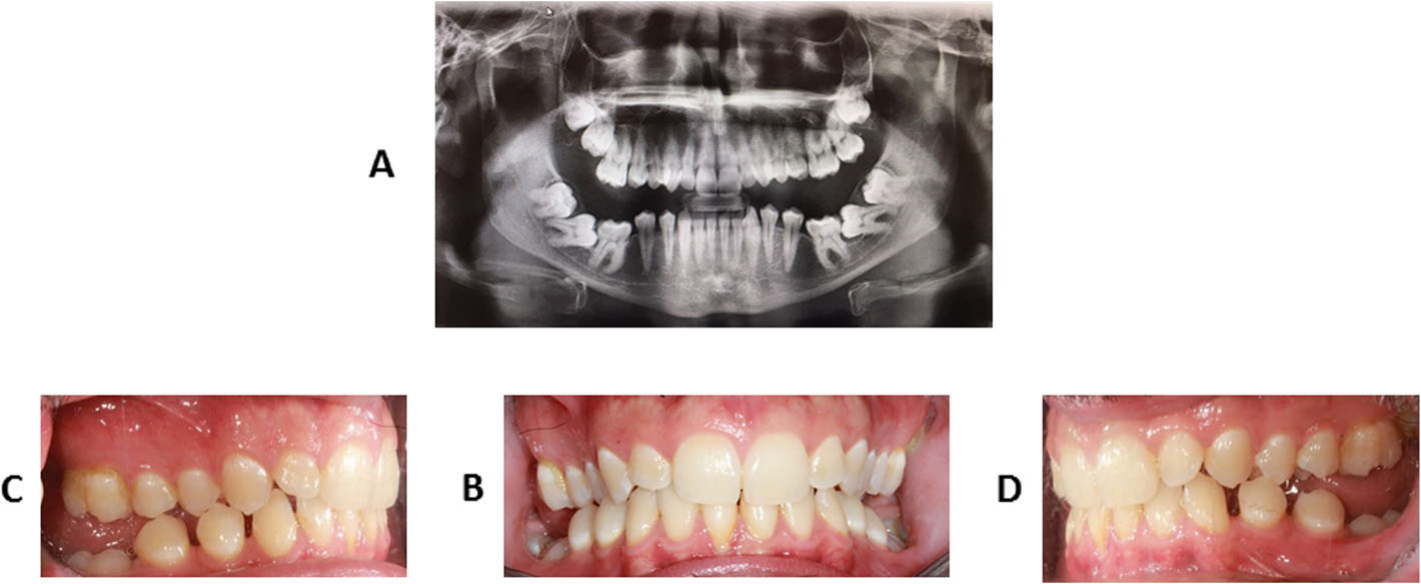
Clinical records of proband: panoramic radiographic analysis (**a**), intraoral frontal view (**b**), intraoral right lateral view (**c**), intraoral left lateral view (**d**)

**Fig. 2:**
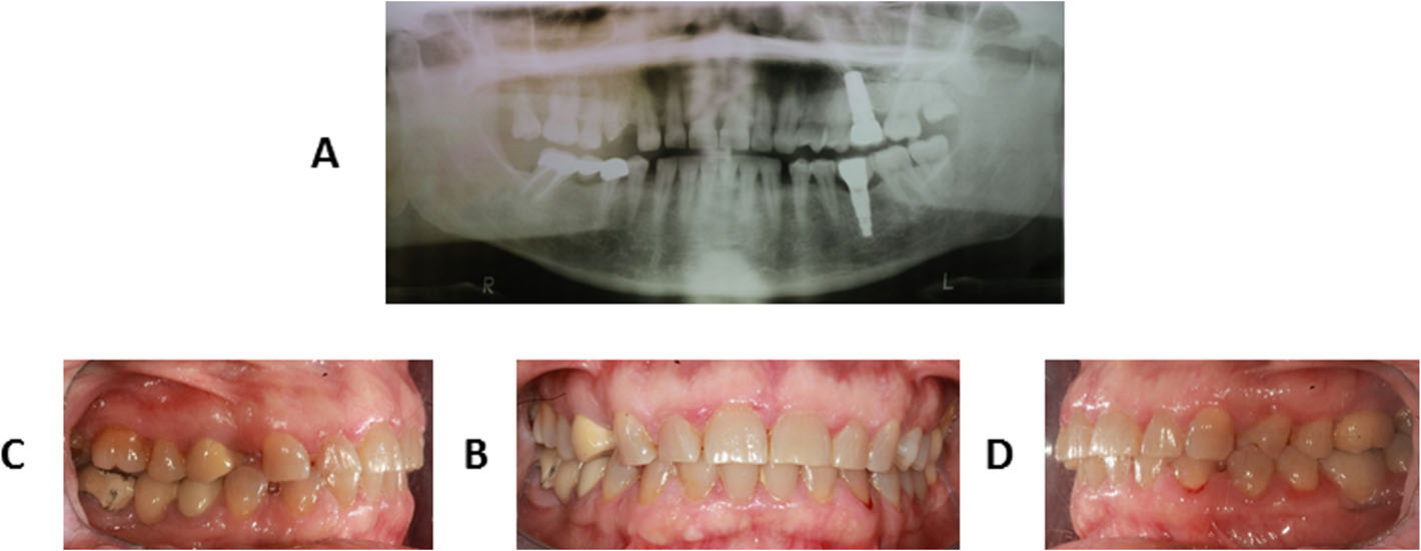
Clinical records of the mother’s proband: panoramic radiographic analysis (**a**), intraoral frontal view (**b**), intraoral right lateral view (**c**), intraoral left lateral view (**d**)

**Fig. 3:**
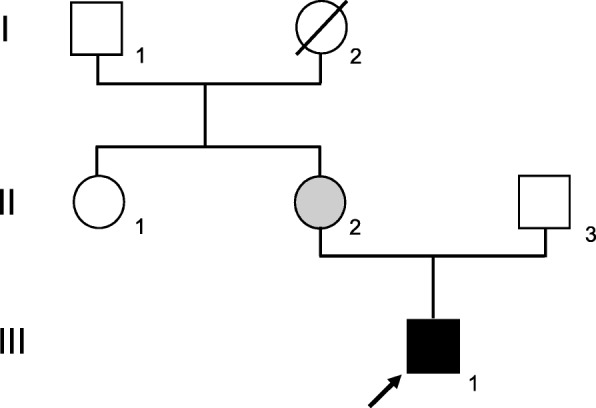
Family tree with affected proband III-1 indicated in black. Open symbols indicate unaffected individuals who do not carry the c.505G > T variant. Individual II-2 (proband’s mother) is indicated in gray since she carries the same variant but does not express the phenotype. Individual I-2 (proband’s maternal grandmother) was dead and her DNA could not be tested

A 26-year-old man was referred to the Orthodontic Clinic of the “A.Gemelli” University hospital in Rome. A clinical examination revealed the presence of a bilateral posterior open bite (Fig. 1a-d). The panoramic radiograph (Fig. 1a) showed affection mainly of the lower arch of the Type I PFE variety [4]. Given the similarity to other PFE cases reported in the literature, genetic counseling was offered to the patient and clinical examination (including panoramic X-rays and photos) of all available relatives - mother (II:2) (Fig. 2a-b-c-d), father (II:3), maternal aunt (II:1) and maternal grandfather (I:1), indicated in Fig. 3 - was conducted at the Dental Clinic of the Fondazione Policlinico “A.Gemelli” IRCCS in Rome, while genetic analysis was carried out at the laboratories of the Institute of Genomic Medicine and the Dental Institute of the Catholic University (UCSC). We also measured the height of the proband and his parents, although there was no evidence of craniofacial and skeletal malformations, in order to test the presence of short stature.

This study was approved by the local Ethics Committee (#UCSC prot. 36,110/10 ID: 565), the procedures followed were in accordance to the Declaration of Helsinki of 1975 and subsequent revisions, and all patients involved provided a written informed consent to participate. Patient’s information were de-identified.

### DNA extraction and sequencing

Cells from saliva and oral mucosa were collected from each available family member by buccal swabs for genetic analysis of the *PTH1R* gene. DNA extraction and purification was carried out using an automatic MagCore Nucleic Acid Extractor. DNA concentration and quality were measured by absorbance at 260 nm and by the ratios of A260 nm/A280 nm. PCR primers were designed using Genamics Expression DNA Sequence Analysis Software and the in Silico-PCR tool provided by the UCSC Genome Browser (http://rohsdb.cmb.usc.edu/GBshape/cgi-bin/hgPcr). Primer sets were designed to include all exons and splice junctions, including a minimum of 50 nucleotides of intron sequence. PCR amplification of the 16 exons was performed using PCR Master Mix (ProMEGA Corporation). PCR cycles consisted of denaturation at 95 °C for 2 min, 38 cycles of 95 °C for 45 s, 55 °C for 45 s, 72 °C for 45 s, and a final elongation at 72 °C for 5 min. PCR products were purified using PCR purification Kit EuroClone ExoStar PCR-plate, directly sequenced on both strands using BigDyeTerminator V3.1 and subsequently resolved on an ABI3130 or ABI3500 Genetic Analyzer (Applied Biosystems, Foster City, USA). Identified variations were confirmed with a new sequencing reaction. NM_000316.2 was used as reference sequence of *PTH1R* gene. Identified variations were confirmed in a new PCR and sequencing reaction.

### In Silico analysis

Sequence data were inspected using Sanger Sequencing and Fragment Analysis Software SeqScape of *Applied* Biosystems (ThermoFisher Scientific). The reference mRNA sequence is NM_000316.3 and variants are indicated starting from the first nucleotide of the coding sequence (+ 1). The reference protein sequence is NP_000307.1 and corresponds to a 593 aminoacid-long receptor with an extracellular N-terminal domain (aa 1–183), 7 transmembrane domains (aa 184–476) and an intracellular C-terminal domain (aa 477–593). *PTH1R* sequence variants were searched in gene-specific databases, such as the Leiden Open Variation database - LOVD (https://databases.lovd.nl/shared/genes/PTH1R) and the NCBI ClinVar database (https://www.ncbi.nlm.nih.gov/clinvar), as well as in general population databases, namely the NCBI Database of Short Genetic Variation – dbSNP (https://www.ncbi.nlm.nih.gov/snp) and the Genome Aggregation Database - GnomAD (https://gnomad.broadinstitute.org/).

### Clinical findings

The proband (III:1) is the only one in the family with a characteristic PFE phenotype: failure of eruption of the first molars, additional posterior teeth involvement, supracrestal position of affected teeth (i.e. the eruption pathway was clear) [2, 3, 11, 12]. If two or more of these clinical and radiographic criteria are found, the probability that PFE is caused by *PTH1R* variants increases [13]. Clinically, the proband (III:1) shows a severe open bite of the lateral-posterior sectors with associated root anomalies (extremely curved roots), especially in molars [13]. Radiographically (Fig. 1a), it is possible to observe the symmetrical infraocclusion of the inferior molars and asymmetrical inclusion of the maxillary molars. In the lower arch we observe the infraocclusion of 3.6 and 4.6 with mesio-inclination and total inclusion of both the second and third molars of both mandibular sides. In the upper arch, instead, the infraocclusion affected the second right molar and both third molars (Fig. 1a-c-d). According to the clinical classification of PFE [4], our patient could be assigned to Type I. On the other hand, all family members were clinically inspected and did not show any clinical sign of PFE. Intraoral pictures and panoramic radiograph of the mother (II:2) are shown in Fig. 2. The proband is 173 cm tall, while his parents are 158 cm (mother II:2) and 171 cm (father II:3).

### Mutational analysis

**Fig. 4:**
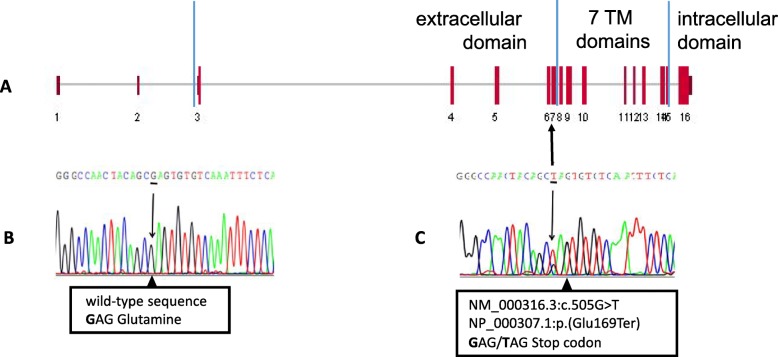
Localization on exon 7 of the nonsense *PTH1R* variant identified in our PFE patient (**a**), nucleotide sequence of a normal individual (**b**), sequence variant of proband III-1 (**c**) with the heterozygous variant G > T shown as a double peak in the electropherogram

A buccal swab was obtained and DNA extracted for all available family members (Fig. 3). Sequencing analysis revealed a novel heterozygous nonsense variant (c.505G > T; p.Glu169Ter) in our PFE patient (III:1), resulting in the insertion of a premature stop codon in exon 7 of the *PTH1R* gene (Fig. 4). This variant is classified as pathogenic since it results in the production of a truncated protein, lacking all 7 transmembrane domains and the intracellular C-terminus. The patient’s variant was then searched in the other family members and it was found only in the patient’s mother (II:2). The maternal grandfather (I:1) did not carry the variant, but we could not test the maternal grandmother (I:2).

## Discussion and conclusions

Genetic analysis of our patient (Fig. 4) revealed a new heterozygous nonsense variant in exon 7 of the *PTH1R* gene (c.505G > T) that introduces a premature stop codon and likely results in the production of a truncated protein (p.Glu169Ter) lacking all transmembrane domains and the cytoplasmic C-terminal domain or possibly causes mRNA degradation through nonsense-mediated decay. This nonsense variant can be considered pathogenic also because it was not found in public databases, both those dedicated to specific gene variants (NCBI ClinVar and LOVD) and those collecting variants of large heterogeneous groups (dbSNP and gnomAD).

However, a single nucleotide variant affecting the same nucleotide (c.505G > A) but resulting in a missense change (p.Glu169Lys) is reported in dbSNP (rs761172480) and was found in 1 out of 246,264 alleles. Codon 169 is also changed in the second position (c.506A > C), resulting in another missense change (p.Glu169Ala), reported in dbSNP (rs1250664469) with a frequency of 1 out of 125,568 alleles, and in the third position (c.507G > A) resulting in a synonymous change (rs763828859) with a frequency of 1 out of 246,266 alleles. It would be very interesting to know if these individuals also present with PFE, but this is impossible since DNA samples belonging to these large datasets have been made anonymous.

By extending the molecular analysis to other family members, we discovered that the unaffected mother had the same variant of the son. This finding underscores the well-known phenomenon of reduced penetrance, which has already been reported in a PFE family [11] and consists in the lack of phenotypic expression of a genetic variant that in other family members results in the expected disorder/condition [14]. Reduced penetrance is an extreme form of variable expressivity and is probably due to the combined action of other genetic, epigenetic as well as microenvironmental factors, converging on the same pathogenic pathway but different for each individual, even in identical twins [15].

We also measured the height of proband and his parents since it is known that *PTH1R* variants are associated to different skeletal syndromes with short stature. Homozygous loss-of-function variants result in Blomstrand chondrodysplasia (OMIM #215045), a perinatally lethal recessive condition characterized by severe limb dwarfism with short ribs, advanced skeletal maturation and severe tooth impaction [16]. In fact, *PTH1R* knockout mice usually die at midgestation [17] but heterozygous animals, which would represent the murine model for PFE, were not described in detail. Two families have been reported with non-lethal homozygous *PTH1R* variants resulting in Eiken syndrome (OMIM #600002) characterized by growth retardation with abnormal bone modeling in hands and feet, delayed skeletal maturation and abnormal persistence of cartilage in the pelvis [18]. Interestingly, the patient with Eiken syndrome reported by Moirangthem et al. [19] has also malposition and impaction of most of his teeth. On the other hand, heterozygous gain-of-function variants have been identified in patients with Jansen metaphyseal chondrodysplasia (OMIM #156400), a dominant condition with PTH1R hyperactivity, short stature, hypercalcemia, hypophosphatemia, extreme disorganization of the metaphyses of the long bones and tooth malposition but mostly normal tooth eruption. PFE patients usually have normal stature, as our proband does (173 cm tall), however we can speculate that his mother’s short stature (158 cm) might be a consequence of *PTH1R* haploinsufficiency and we suggest that anthropometric measures of PFE family members be systematically collected during clinical examination.

A genetic diagnosis aimed at excluding or confirming the presence of pathogenic variants in the *PTH1R* gene should be carried out in every patient with suspected PFE to help confirm the diagnosis and to avoid failure of orthodontic intervention. In this report we underlined the importance of a careful genotype-phenotype correlation and we extended the clinical evaluation to all available family members, including anthropometric measurements. The nonsense variant found in our index case (c.505G > T;p.Glu169Ter), as in previous instances, has never been reported before and was identified in the unaffected mother. This confirms that incomplete penetrance, as well as variable expressivity, is a common event in PFE. Phenotypic variation is due to genetic, epigenetic and environmental modifiers, although most physiopathological pathways implicated in PFE remain unknown. Clinical and genetic analysis of familial cases will assist our understanding of the cellular mechanisms underlying this condition and will pave the way to innovative pharmacological treatments [20].

## Data Availability

DNA of family members and original sequence electropherograms are available upon request.

## Abbreviations

MFE: Mechanical Failure of Eruption
PCR: Polymerase Chain Reaction
PFE: Primary Failure of Eruption
*PTH1R*: Parathyroid Hormone 1 Receptor
